# Community wide electronic distribution of summary health care utilization data

**DOI:** 10.1186/1472-6947-6-17

**Published:** 2006-03-20

**Authors:** Ronald J Lagoe, Gert P Westert

**Affiliations:** 1 Hospital Executive Council, Syracuse, New York, USA; 2 National Institute of Public Health, and the Environment Bilthoven, Netherlands

## Abstract

**Background:**

In recent years, the use of digital technology has supported widespread sharing of electronic health care data. Although this approach holds considerable promise, it promises to be a complicated and expensive undertaking. This study described the development and implementation of a community wide system for electronic sharing of summary health care utilization data.

**Methods:**

The development of the community wide data system focused on the following objectives: ongoing monitoring of the health care system, evaluation of community wide individual provider initiatives, identification and development of new initiatives.

The system focused on the sharing of data related to hospital acute care, emergency medical services, long term care, and mental health. It was based on the daily distribution of reports among all health care providers related to these services.

**Results:**

The development of the summary reports concerning health care utilization produced a system wide view of health care in Syracuse, New York on a daily basis. It was not possible to isolate the results of these reports because of the impact of specific projects and other factors. At the same time, the reports were associated with reduction of hospital inpatient stays, improvement of access to hospital emergency departments, reductions in stays for patients discharged to nursing homes, and increased access of mental health patients to hospital inpatient units.

**Conclusion:**

The implementation of the system demonstrated that summary electronic utilization data could provide daily information that would support the improvement of health care outcomes and efficiency. This approach could be implemented in a simple, direct manner with minimal expenses.

## Background

In recent years, interest in the development and dissemination of health care data has increased within the United States. This interest has been fueled by the widespread use of digital technology to share information and the production of electronic medical record data by providers of care [1-3].

For many years, discussions of the use of electronic health care data have focused on broad objectives and program outlines. These conversations recognized that the widespread implementation of electronic systems would be a complicated and extremely expensive undertaking [4]. The development of presidential interest and priorities concerning electronic medical records could change this situation. At the same time, the costs of operating an electronic data system will outstrip those required for development. With the Medicare and Medicaid programs competing for scarce federal and state dollars, this funding could be difficult to generate [4-6].

In these circumstances, incremental approaches to the development and distribution of electronic health care data have become more important. Research has already demonstrated the benefits of electronic documentation for record review, prescription and test ordering, and care management [2,7]. Hospitals and physician groups have implemented electronic systems for sharing health care data [8].

This study describes the development and implementation of a community wide system for electronic sharing of summary health care utilization data in Syracuse, New York. It demonstrates how this system is providing simple, low cost data concerning acute care, emergency medical services, long term care, and mental health programs that have improved the efficiency of the community's health system.

## Results

The development of the summary reports concerning health care utilization in the Syracuse metropolitan area produced a system wide view of the health care system on a daily basis. This perspective included major acute hospital programs, emergency medical services, long term care facilities, and mental health emergency and inpatient resources. These reports provided information for ongoing monitoring of health care utilization on a community wide and institution specific basis. They also provided support for development and evaluation of the impact of specific programs.

It was not possible to isolate the results of these summary utilization reports on the community because of the impacts of specific projects, secular trends, and other factors. For this reason, the opportunity did not exist for evaluation of the impact of data feedback alone on the system. The authors acknowledge and emphasize the limitations of the summary data from this perspective.

The summary utilization reports were employed to monitor a number of existing health care programs in the community. The reports also encouraged the development of additional programs to improve health care outcomes and utilization in the Syracuse metropolitan area.

These programs included the following.

Length of stay reduction programs existed at the Syracuse hospitals before development of the inpatient census report. The report supported these efforts by encouraging a daily focus on census and turnover levels.

The emergency medical services report was implemented at the same time as a number of internal hospital programs to improve the efficiency of treatment in hospital emergency departments. The report has been used on an ongoing basis to monitor the implementation of these programs.

An important component of hospital length of stay reduction programs has been expediting the movement of patients between hospitals and nursing homes. The long term care report has contributed to these efforts by improving the placement process for difficult to place patients.

The mental health report was implemented at the time of the development of multihospital management of the community wide psychiatric emergency department. The report has supported this effort by improving the tracking of mental health utilization.

The distribution of the summary reports supported changes in several areas of health care utilization in the Syracuse area. These developments resulted from a combination of the data distribution and the implementation of specific projects. Data concerning examples of these changes are summarized in Table 3.

Some of the most notable improvements in efficiency identified in Table 3 concerned inpatient lengths of stay, which has been tracked on the daily reports through admissions and discharges for medical-surgical beds, as well as through census levels. These developments resulted from hospital efforts to reduce stays through specific programs involving physicians, ancillary services such as testing, and discharges to long term care services. The daily information in the reports supported ongoing evaluation of these programs. Between 2000 and 2004, medical-surgical mean stays in the Syracuse hospitals declined by 12.9 percent, 14.0 percent for adult medicine and 12.0 percent for adult surgery. This was the highest rate of decline for any New York State metropolitan area. It resulted in the elimination of over 28,000 inpatient days.

The data in Table 3 also demonstrate improvements in access to hospital emergency departments related to the distribution of the daily reports. The daily information concerning emergency medical services made an important contribution to these results. The report has made it possible for hospital managers to evaluate the impact of operational initiatives on an immediate basis both within their institutions and throughout the system. This progress was impressive. As the total number of hospital emergency department visits increased by 12.8 percent and the total ambulance volume expanded by 21.3 percent, hours on ambulance diversion declined by 48.0 percent.

The introduction of the long term care report was also associated with improvements in the efficiency of the movement of patients between hospitals and nursing homes. These reports made it possible to monitor the impact of the program to increase difficult to place admissions to nursing homes and the subacute programs on a daily basis. Through the combined impact of these programs and the data, the rate of decline of mean hospital stays for patients discharged to nursing homes increased by 24.3 percent between 2000 and 2004. During the same period, the mean length of stay for hospital discharges to nursing homes declined by 19.5 percent between 2002 and 2004. At the same time, the size of the hospital non acute population declined by 68 percent.

The data from the psychiatric emergency department also suggest that, in fewer than two years of distribution, the mental health report has also supported positive change. Prior to the distribution of this report, system wide data were not available on a daily basis for mental health programs. The report made it possible to monitor inpatient programs on a daily basis and discouraged refusal of admission referrals from the psychiatric emergency department. Since its introduction, in 2003, the number of refusals of psychiatric emergency department referrals by hospital psychiatric units has declined by over 60 percent. In the same period, the amount of time the program was at maximum capacity has fallen by approximately 50 percent.

Beyond the impact of the summary reports on health care utilization in the community, this information has also contributed to the monitoring of health and related areas. The emergency medical reports are provided on a daily basis to the Onondaga County Department of Health, which has used them to track emergency department utilization through the respiratory disease season each year. It has contributed to decision making concerning the distribution of influenza vaccine and other public health initiatives. The acute care and long term care reports are provided to the New York State Department of Health, which has employed them to monitor access to hospitals and nursing homes. These reports have contributed to the implementation of extraordinary nursing home beds in the system at times of high occupancy in hospitals. The mental health reports are sent to the Onondaga County Department of Mental Health, which has used them to track patients being sent outside the community. These reports contributed to the approval of additional mental health beds in the community by the New York State Office of Mental Health.

## Discussion

The development and distribution of summary health care utilization data in the metropolitan area of Syracuse, New York has been developed over a five year time period. It has involved all major components of the community's health care system in a daily review of the use and efficiency of acute care, emergency medical, long term care, and mental health services.

It must be emphasized that this system has been a limited approach to monitoring health care utilization and stimulating positive change. Its impact could not be evaluated apart from the results of specific initiatives within the health care system.

This program has been successful for a number of reasons. A major factor has been its simplicity and low cost. Using reports from existing sources and electronic mail, the daily distribution has required limited resources and staff time, even at the Hospital Executive Council which distributes the reports.

Another factor behind the success of this program has been its relationship with current professional culture. This culture is quantitative to the point of being 'nerdy'. Almost everyone wants and uses statistics. In this environment, summary health care data, updated on a daily basis, has no difficulty generating interest.

The development and use of these data have also been successful because they are consistent with the competitive nature of health care. In the daily acute care, emergency medical services, long term care, and mental health reports, major providers can check the status of their competitors throughout the system. This aspect of the reports has encouraged benchmarking concerning hospital inpatient turnover, ambulance diversion hours, nursing home occupancy, and other indicators. The availability of monthly reports has provided additional information for the competitive market.

Perhaps the most important factor in the success of this system has been its use of electronic mail, rather than a website, for distribution. Electronic mail has ensured that each participant receives each report at the beginning of every business day. Access to this information is not an option that is exercised by using a website and drilling down, the data are placed literally in the face of every user. Only the delete button will remove the information.

Sustained by these advantages the system for electronic distribution of health care utilization data in Syracuse has supported notable improvements in the efficiency of the local health care system. Because other factors have also contributed to these improvements, it was not possible to isolate the impact of the electronic data system. This mechanism has, however, provided a daily resource for monitoring the use of health care throughout the community.

In this manner, the electronic data system has stimulated creativity concerning improvements in services and relationships among them, as well as providing an instrument for tracking those changes. The impact of this system on the community continues to play out.

All of these factors suggest that the system for electronic distribution of summary health care utilization data has much to offer to other communities. As a simple, direct model, it provides an inexpensive mechanism for monitoring that can be implemented and effective within a short period of time.

## Methods

This study described the implementation and impact of a community wide health care data system in the metropolitan area of Syracuse, New York. This area includes the City of Syracuse and Onondaga County, with an estimated population of 451,366 (2005). The four local hospitals serve as the regional referral center for the Central New York Health Service Area, which includes a population of 1,420,286 (2005).

The community wide data are based on four Syracuse hospitals. These facilities include University Hospital of the State University of New York Upstate Medical University, a medical school hospital with 16,943 discharges (2005), two large community acute care facilities with some tertiary services – St. Joseph's Hospital Health Center with 24,680 discharges (2005)and Crouse Hospital with 21,992 discharges (2005), and another general hospital – Community-General Hospital with 10,510 discharges (2005). Historically, these competing hospitals have developed and implemented numerous cooperative programs through their joint planning organization, the Hospital Executive Council.

The community wide data base also includes the emergency medical services system of the Syracuse area. This network includes thirty two ambulances services which provide care to Onondaga County. The largest of these is Rural Metro Medical Services, which covers the City of Syracuse. The network also includes an additional thirty five ambulance units, which serve the four counties contiguous to Onondaga County. Each of the general hospitals operates an emergency department. The department at University Hospital serves as the region's trauma center. During 2005, the four hospitals provided a total of 181,851 emergency department visits including 25,257 at Community-General Hospital, 54,958 at Crouse Hospital, 50,990 at St. Joseph's Hospital Health Center, and 50,646 at University Hospital.

The data system for the Syracuse metropolitan area also includes thirteen nursing homes of Onondaga County. These facilities contain a total of 3,107 long term care beds. Approximately half of the total capacity is contained in three facilities, Loretto (535 beds), VanDuyn Home and Hospital (526 beds), and James Square Health and Rehabilitation Center (455 beds).

The data system also includes the hospital and emergency psychiatric programs in the Syracuse metropolitan area. Hutchings Psychiatric Center, a State owned facility, operates a total of 120 inpatient beds. Community – General Hospital, St. Joseph's Hospital Health Center, and University Hospital maintain an additional 80 psychiatric beds. Beginning in 2003, the three general hospitals assumed joint control of the Comprehensive Emergency Psychiatric Program (CPEP), the area's only mental health emergency department. This program had previously been operated by St. Joseph's Hospital Health Center.

The development of the community wide utilization data system for the City of Syracuse and Onondaga County focused on the development and distribution of summary information concerning use of the health care system. This process was based on provision of data to support the following objectives [9].

Ongoing monitoring of the system

Evaluation of community wide and individual provider initiatives

Identification and development of new initiatives

### Acute care report

The initial focus of data development was acute care, especially adult medicine and surgery. This activity was stimulated by a recognition among the Syracuse hospitals of the need for increased efficiency in the delivery of inpatient care and a belief that daily sharing of inpatient data could contribute to this objective. It was decided that the following information should be reported by each hospital to the Hospital Executive Council on a daily basis, by the admissions department of each hospital.

Medical Surgical Daily Census

Adult Critical Care Daily Census

Pediatric Daily Census

Obstetric Daily Census

Mental Health Daily Census

Total Acute Average Daily Census Excluding Well Newborns and Rehabilitation

Medical-Surgical Daily Admissions

Medical-Surgical Daily Discharges

Each daily report included these indicators for the previous seven days for each hospital and the combined system. A sample of these reports, containing community wide data, is summarized in Table 1. In addition, the reports also include data for each of the hospitals. [See Additional File 1] for a sample of the complete report.

**Table 1:** Hospital Inpatient Census Medical/Surgical and Intensive/Cardiac Care Units Syracuse Hospitals.

	Census			Medical/Surgical & ICU/CCU			Emergency Department Visits
	Medical/Surgical	Adult ICU/CCU	Total Excluding Newborn	Admissions	Discharges	Turnover	
Totals							
1-Jan	581	71	854	130	124	-6	509
2-Jan	598	65	879	70	57	-13	442
3-Jan	609	75	924	159	144	-15	408
4-Jan	658	83	979	172	130	-42	417
5-Jan	657	83	983	155	150	-5	482
6-Jan	654	86	975	83	93	10	452
7-Jan	648	87	962	74	73	-1	473
Mean	629	79	937	843	771	-72	3,183

The acute care report has been distributed each business day by 9:00 a.m. from the Hospital Executive Council office beginning in December 1999. Recipients have included approximately 100 individuals, including the chief executive officers, vice presidents, and other administrators of the Syracuse hospitals.

### Emergency medical services

The production of the daily acute care reports for the Syracuse metropolitan area in December 1999 was paralleled by the development of a report concerning emergency medical services. Interest in this report was initially stimulated by eliminating overcrowding of emergency departments among the Syracuse hospitals.

The development of the emergency medical services report was expedited by the operation of a single mechanism for online monitoring of ambulance diversion and ambulance distribution in Syracuse, the EMSystem. This software program, developed and distributed by Infinity Health Care of Milwaukee, Wisconsin, linked the four Syracuse hospitals and the two organizations responsible for dispatching ambulances, the Onondaga County 911 Center and Rural Metro Medical Services.

Hospital and emergency medical services representatives decided that this report should include the following information for the combined system and each of the Syracuse hospitals.

Daily Ambulance Transports – Total, On Diversion, and Open

Daily Ambulance Diversion Hours

Daily Emergency Department Visits

The format of the emergency medical services report for community wide data is summarized in Table 2. The report also includes the same information for individual hospitals. [See Additional File 1] for a sample of the complete report.

**Table 2:** Hospital Emergency Department Ambulance Transports Received Syracuse Hospitals.

	On Diversion			Open			Total Transports	Total Transports Per Hour	Emergency Department Visits
	Number of Hours	Transports Received	Transports Per Hour	Number of Hours	Transports Received	Transports Per Hour			
Total									
1-Jan	0.0	0	-	96.0	100	1.0	100	4.2	509
2-Jan	4.0	1	0.3	92.0	106	1.2	107	4.5	442
3-Jan	11.6	6	0.5	84.4	86	1.0	92	3.8	408
4-Jan	5.1	6	1.2	90.9	99	1.1	105	4.4	417
5-Jan	16.2	3	0.2	79.8	87	1.1	90	3.8	482
6-Jan	5.1	7	1.4	90.9	80	0.9	87	3.6	452
7-Jan	22.8	18	0.8	73.2	106	1.4	124	5.2	473
Total	64.8	41	0.6	607.2	664	1.1	705	4.2	3,183

**Table 3:** Health Care Utilization Indicators Syracuse, New York Metropolitan Area 2000 – 2004.

Indicator	2000	2001	2002	2003	2004
Inpatient					
Adult Medicine Mean Length of Stay	5.7	5.7	5.4	5.1	4.9
Adult Surgery Mean Length of Stay	6.9	6.8	6.8	6.3	6.1
Medical/Surgical Mean Length of Stay	6.2	6.2	6.0	5.7	5.4
Emergency Department					
Visits	154,857	167,435	170,063	173,317	174,741
Ambulance Transports	31,987	34,460	36,492	36,987	38,811
Diversion Hours	11,398	8,677	5,588	6,548	5,928
LOS for Discharges to Nursing Homes	12.1	12.0	11.3	10.3	9.1
Total Admissions to Nursing Homes	4,554	4,998	4,720	5,178	5,662
Mean Alternate Care (Non Acute) Census	35.3	33.2	28.9	18.6	11.3
CPEP					
Refusal of CPEP Referrals	205	198	241	91	99
Time at Maximum Capacity	624	568	572	364	335

### Long term care

The development of summary reports concerning long term care services in the Syracuse metropolitan area was stimulated by the same interest in improving the efficiency of system wide utilization that supported the initiation of the acute care and emergency medical services reports. Historically, the Syracuse hospitals had worked with the twelve nursing homes and two major home health agencies of Onondaga County to expedite the movement of patients between these levels of care.

The distribution of the acute care and emergency medical services reports during 2000 and 2001 stimulated interest in electronic mail distribution of summary utilization data concerning long term care services, as well use of this medium for transmission of alternate care data. This information included the following indicators.

Unoccupied Beds by Nursing Home

Candidates for Nursing Home Placement by Hospital

Daily reports concerning unoccupied nursing home beds were developed for all administrators concerned with utilization management, each individual hospital care manager, nursing home administrator, home health agency administrator, and nursing home admissions director. In addition to this report, the hospitals distribute to all area nursing homes and home health agencies a list of individuals identified as difficult to place. This report is sent twice each week. [See Additional File 1] for a sample of the complete report.

### Mental health

The daily distribution of summary reports concerning acute care, emergency medical services, and long term care in the Syracuse metropolitan area provided the opportunity for efforts related to mental health services. During the first quarter of 2003, these efforts were supported by the movement of the community's only psychiatric emergency department, the Community Psychiatric Emergency Department (CPEP) from sponsorship by a single hospital to multihospital control.

With this process came an interest in tracking psychiatric inpatient and emergency utilization throughout the community. The following indicators were identified for this report.

Census by Hospital

Admissions and Discharges by Hospital

Psychiatric Emergency Department Census

Psychiatric Emergency Department Referrals by Hospital

Psychiatric Emergency Department Time at Maximum Capacity

Refusals of Referrals from Psychiatric Emergency Department by Hospital and Reason

Like the acute care, emergency medical services, and long term care reports, the mental health report was scheduled for distribution every business day by electronic mail. This format also includes utilization for adult and psychiatric services at Hutchings Psychiatric Center, a large, state operated mental health hospital located in the center of Syracuse. [See Additional File 1] for a sample of the complete report.

### Monthly reports

In addition to the daily reports, the Hospital Executive Council also develops and distributes data concerning acute care, emergency medical services, long term care, and mental health services on a monthly basis. These reports are developed after the completion of coding and are based on consistent definitions applied to patient specific information.

## Competing interests

The author(s) declare that they have no competing interests.

## Authors' contributions

R.J.L. coordinated the development of the data system in Syracuse, New York, USA. He is responsible for monitoring and supervising the operation of the data system. He was partially responsible for developing the data included in this study.

G.P.W. participated in the development of the data system. He provided suggestions for types of data to be included in the system. He also shared responsibility for the development of the data analysis in the manuscript.

## Pre-publication history

The pre-publication history for this paper can be accessed here:



## Supplementary Material

Additional File 1 Formats for Daily Utilization Reports – This file includes samples of each of the four daily reports identified in the Methods Section. These formats were used in the procedures described in the study. Additional information concerning these formats is available from the authors. Click here for file
